# Scribble is required for normal epithelial cell–cell contacts and lumen morphogenesis in the mammalian lung

**DOI:** 10.1016/j.ydbio.2012.11.012

**Published:** 2013-01-15

**Authors:** Laura L. Yates, Carsten Schnatwinkel, Lee Hazelwood, Lauren Chessum, Anju Paudyal, Helen Hilton, M. Rosario Romero, Jonathan Wilde, Debora Bogani, Jeremy Sanderson, Caroline Formstone, Jennifer N. Murdoch, Lee A. Niswander, Andy Greenfield, Charlotte H. Dean

**Affiliations:** a Mammalian Genetics Unit, Medical Research Council, Harwell, UK; b Howard Hughes Medical Institute, Dept of Pediatrics, Section of Developmental Biology, University of Colorado Denver School of Medicine and Children's Hospital Colorado, Aurora, CO, USA; c MRC Centre for Developmental Neurobiology, New Hunts House, Kings College London, London SE1 1UL, UK; d School of Biological Sciences, Royal Holloway, University of London, Egham, Surrey, TW20 0EX, UK

**Keywords:** Scribble, Lung, Branching morphogenesis, Lumen, PCP, Polarity

## Abstract

During lung development, proper epithelial cell arrangements are critical for the formation of an arborized network of tubes. Each tube requires a lumen, the diameter of which must be tightly regulated to enable optimal lung function. Lung branching and lumen morphogenesis require close epithelial cell–cell contacts that are maintained as a result of adherens junctions, tight junctions and by intact apical–basal (A/B) polarity. However, the molecular mechanisms that maintain epithelial cohesion and lumen diameter in the mammalian lung are unknown. Here we show that Scribble, a protein implicated in planar cell polarity (PCP) signalling, is necessary for normal lung morphogenesis. Lungs of the *Scrib* mouse mutant *Circletail* (*Crc*) are abnormally shaped with fewer airways, and these airways often lack a visible, ‘open’ lumen. Mechanistically we show that *Scrib* genetically interacts with the core PCP gene *Vangl2* in the developing lung and that the distribution of PCP pathway proteins and Rho mediated cytoskeletal modification is perturbed in *Scrib*^*Crc/Crc*^ lungs. However A/B polarity, which is disrupted in *Drosophila Scrib* mutants, is largely unaffected. Notably, we find that Scrib mediates functions not attributed to other PCP proteins in the lung. Specifically, Scrib localises to both adherens and tight junctions of lung epithelia and knockdown of Scrib in lung explants and organotypic cultures leads to reduced cohesion of lung epithelial cells. Live imaging of Scrib knockdown lungs shows that Scrib does not affect bud bifurcation, as previously shown for the PCP protein Celsr1, but is required to maintain epithelial cohesion. To understand the mechanism leading to reduced cell–cell association, we show that Scrib associates with β-catenin in embryonic lung and the sub-cellular distribution of adherens and tight junction proteins is perturbed in mutant lung epithelia. Our data reveal that *Scrib* is required for normal lung epithelial organisation and lumen morphogenesis by maintaining cell–cell contacts. Thus we reveal novel and important roles for *Scrib* in lung development operating via the PCP pathway, and in regulating junctional complexes and cell cohesion.

## Highlights

► Scrib signals via the PCP pathway to regulate lung branching morphogenesis. ► Knockdown of Scrib leads to reduced cohesion of lung epithelial cells. ► Scrib is required for normal epithelial organisation and lumen morphogenesis in embryonic lung.

## Introduction

Lung organogenesis involves the formation of a network of epithelial tubes with an extensive surface area to support postnatal respiration. New tubes are formed by budding of groups of polarised epithelial cells from an existing tube (Andrew and Ewald, 2009; Hogan and Kolodziej, 2002; Nelson, 2003). In the mouse, the spatial pattern of lung branches is remarkably stereotypical and is generated by three modes of local branching, named domain branching and planar and orthogonal bifurcation (Metzger et al., 2008).

Establishment and maintenance of a central lumen within each epithelial tube is a key step in tubulogenesis that allows efficient transport of liquids or gases (Andrew and Ewald, 2009; Chung and Andrew, 2008; Paul et al., 2003). Moreover, lumen diameter must be carefully regulated to facilitate optimal organ function (Datta et al., 2011). Current understanding of the molecular mechanisms of mammalian lumen morphogenesis is limited, yet disrupted lumen diameter is a feature of many human diseases such as polycystic kidney disease, hypertension and ischemic injury. In the lung, understanding the mechanisms used to establish and maintain lumen size may be important for treatment of cystadenomatoid malformations, pulmonary hypertension and even asthma, in which narrowing of the upper airways occurs.

Preserving sufficient lumen diameter requires maintenance of close contacts between epithelial cells through adherens junctions and tight junctions (Chung and Andrew, 2008; Lubarsky and Krasnow, 2003; Martin-Belmonte and Mostov, 2008). Formation of these junctional complexes is underpinned by the establishment of A/B polarity, characterised by similarly aligned cells with their basal sides immediately adjacent to the basement membrane and their apical sides adjacent to the lumen (Martin-Belmonte and Mostov, 2008; Martin-Belmonte et al., 2008). In lung branching morphogenesis, lumina are not formed *de novo*, but instead, new tubes arise from clefting or budding of existing tubes containing polarised epithelial cells so that the lumen of the new bud/branch is continuous with the lumen of the existing branch (Andrew and Ewald, 2009; Chung and Andrew, 2008; Hogan and Kolodziej, 2002). Initially, the lumen has a narrow diameter and this subsequently widens as the tube matures to its optimal size (C.D. unpublished observations). Although it is known that establishment of ion channels and secretion of fluid into the luminal space *in utero* play a role in regulating lung lumen diameter (Wilson et al., 2007), epithelial cells must first establish and preserve A/B polarity, undergoing considerable dynamic cell shape changes, mediated by the cytoskeleton, in order to adopt the morphology necessary to encompass a lumen. Moreover, it is essential that strong cell–cell interactions be maintained, to preserve the luminal space (Andrew and Ewald, 2009).

Scribble is a large cytoplasmic protein containing multiple domains including 4 PDZ domains (Bilder and Perrimon, 2000; Nakagawa and Huibregtse, 2000; Nakagawa et al., 2004). In *Drosophila*, Scrib is initially located at the basolateral membranes of epithelial cells and later in development becomes more restricted to septate junctions (Bilder and Perrimon, 2000). In mammalian cells *in vitro*, Scrib is observed at the plasma membrane where it has been shown to influence certain adherens and tight junction proteins including E-cadherin, β-catenin, ZO-1 and ZO-2 (Ivanov et al., 2010a; Metais et al., 2005; Navarro et al., 2005; Qin et al., 2005; Yoshihara et al., 2011). However these studies have reported divergent data concerning the interaction of Scrib with junctional proteins and to date, the mechanism is still unclear. It is notable that mice have only one *Scrib* gene, in contrast to many of the major apical–basal and planar polarity proteins which are represented by multiple family members.

Scribble acts as a tumour suppressor (Etienne-Manneville, 2009): *Drosophila Scrib* null mutants exhibit disorganization of epithelial tissues, leading to neoplastic growth and multilayering of epithelial cells (Bilder et al., 2000; Bilder and Perrimon, 2000) and *SCRIB* expression is decreased in a number of human cancers (Gardiol et al., 2006; Ivanov et al., 2010a; Navarro et al., 2005; Pearson et al., 2011; Thomas et al., 2005). Related to its tumour suppressor role, *Scrib* has been shown to play a part in maintaining contacts between epithelial cells (Dow et al., 2007; Qin et al., 2005) and in regulating the assembly of tight junctions in intestinal epithelium (Ivanov et al., 2010a).

*Drosophila Scrib* is required to maintain A/B polarity as part of a polarity protein complex, along with lethal giant larvae (Lgl) and discs large (Dlg); knockdown of Scrib disrupts *Drosophila* A/B polarity (Humbert et al., 2008). In contrast, most mammalian investigations have shown that *Scrib* operates within the PCP pathway, to regulate planar cell polarity (Montcouquiol and Kelley, 2003; Montcouquiol et al., 2003; Murdoch et al., 2003; Vandenberg and Sassoon, 2009; Wansleeben et al., 2010). In addition, *Scrib* has previously been shown to genetically interact with *Vangl2*; double heterozygotes exhibit defects such as craniorachischisis and disrupted stereociliary bundle orientation that are indicative of planar polarity pathway defects (Montcouquiol et al., 2003; Murdoch et al., 2001). Interestingly, a recent study revealed that *Scrib* does play a role in establishing PCP in *Drosophila*, in addition to its well-characterized role in A/B polarity (Courbard et al., 2009), and one study demonstrated mild A/B polarity defects in mammary epithelial cells (Courbard et al., 2009; Zhan et al., 2008). In fact, *Drosophila* studies show that PCP and A/B polarity pathways are closely linked at the molecular level (Courbard et al., 2009; Djiane et al., 2005) and it may be that many epithelial tissues require both A/B polarisation and planar polarisation for optimal organisation and function.

Given the known functions of *Scrib* in cell polarity and epithelial organisation along with our previous studies showing the importance of PCP proteins in lung development, we investigated lung morphogenesis in the *Scrib* mouse mutant *Circletail*. Here we show that *Scrib*^*Crc/Crc*^ lungs are irregularly shaped and contain fewer epithelial branches. Branches are comprised of disorganised epithelial cells with a narrow lumen diameter or, frequently, no lumen at all. Molecular analysis reveals no overt disruption to A/B polarity but significant perturbation of the actin–myosin cytoskeleton. Moreover, there are reduced levels of active RhoA and altered localisation of the PCP proteins Vangl2 and Celsr1, consistent with Scrib operating within the PCP pathway during lung development. We also show a genetic interaction between *Scrib* and the core PCP gene *Vangl2* in embryonic lung. Additionally, our studies reveal unique roles for Scrib that have not been attributed to other previously studied PCP genes in lung development. Time-lapse imaging of lung branching morphogenesis in the presence of Scrib antisense morpholinos reveals reduced cohesion between epithelial cells. Moreover, *in vivo*, Scrib interacts with the adherens protein β-catenin in lung tissue. Further functional studies show mislocalisation of some tight and adherens junction proteins in *Scrib*^*Crc/Crc*^ lungs. These defects in epithelial tubulogenesis are mimicked *in vitro*, where Scrib knockdown in organotypic cultures results in cysts comprised of disordered cells, small or absent lumina and disrupted sub-cellular localisation of β-catenin, ZO-2 and ZO-1. Our data reveal the importance of *Scrib* function during normal mammalian lung tubulogenesis, particularly in sustaining lumen diameter.

## Materials and methods

### Mouse strains and genotyping

*Scrib*^*Crc*^ mice, originally described in Rachel et al.( 2000) were maintained on a C3H/HeH background. *Scrib*^*Crc*^ mice carry a single base insertion (Murdoch et al., 2003) and were genotyped by PCR amplification of flanking SNPs at 74.88 and 76 Mb (primer sequences available on request) with an annealing temperature of 62 °C and 38 cycles, followed by pyrosequencing. Using limb morphology as an indicator of developmental age, we found no evidence of developmental delay in homozygous mutant embryos compared to wildtype.

### Morphometric analysis

Transverse sections of E14.5 or E18.5 left lung lobes stained with H&E were used to measure the width and number of airways. Sections were obtained from equivalent levels along the rostral–caudal lung axis. Airway widths were calculated by measuring the diameter of each airway at its widest point, using the scale bar tool from Zeiss Axiovision software. The mean width of airways was then determined from all of the airways in a field of view taken from 6 sections per sample and using an n of 4 individual samples per genotype. The number of airways was determined by counting the total number of airways within a section from 6 sections per lung sample from an n of 4 individual embryos per genotype.

The ratios of lumen area *versus* total epithelial airway area at E14.5 were calculated using Velocity 5.4.1 software. Calculations were obtained by measuring the airways present in 5 mutant and 5 wildtype H&E stained transverse lung sections taken from equivalent regions along the rostral–caudal axis of at least 4 separate lungs. The data presented show the percentage of the airway occupied by a lumen. For cell density calculations, the ratio of the number of nuclei: basal airway perimeter in μM was calculated for 33 wildtype and 34 mutant airways from equivalent regions of 4 individual lungs per genotype.

### Antibodies, immunostaining and immunoblotting

Four micrometer paraffin sections or 10 μm cryosections were stained with haematoxylin and eosin or immunostained using antibodies as previously described (Dean et al., 2005; Yates et al., 2010) or with antibodies to: Celsr1 1:1000 (Formstone et al., 2010); Scrib 1:200 (C-20 or H-300); aquaporin-5 1:400; ZO-2 1:200, CC-10, 1:1000; using antigen retrieval, Santa Cruz; ZO-1 1:125; rhodamine phalloidin 1:40, Invitrogen; E-cadherin 1:1000, Cell Signaling Technology; Claudin-18 Zymed Laboratories. Incubations were overnight at 4 °C.

Immunoblotting was carried out using 10 μg/lane of E13.5 wildtype and *Scrib*^*Crc/Crc*^ (*n*=at least 3 per genotype) whole lung protein extract using antibodies as above or to phospho-PKC 1:1000, Cell signalling technology, GAPDH-HRP 1:3000 or anti-β tubulin 1:10000 for loading controls.

### Immunoprecipitation and Rho pull-down assay

Whole lung extracts were prepared by lysing the tissue in 50 mM Tris pH8, 150 mM NaCl, 1% NP-40 substitute, Complete EDTA-free protease inhibitors (Roche) and PhosSTOP phosphatase inhibitors (Roche). Immunoprecipitation of β-catenin complexes from whole lung extracts was performed using Dynabeads-Protein G Immunoprecipitation kit (Invitrogen) according to the manufacturer's instructions. 4 μg of anti-β-catenin antibody (Abcam ab16051) was incubated with 50 μl of Dynabeads-Protein G and allowed to bind for 10 min. After washing, antibody/beads complexes were added to 1 mg of tissue extract and incubated for 2 h at room temperature. Immunocomplexes were then washed and eluted in electrophoresis LDS loading buffer (Invitrogen).

Activated Rho was pulled down from wildtype and *Scrib*^*Crc/Cr*c^ lungs using the Active Rho pull-down and detection kit (Pierce) with modifications as described (Karner et al., 2009), followed by immunoblotting for RhoA using 8 μl/lane of E13.5 wildtype and *Scrib*^*Crc/Crc*^ whole lung protein extract with total RhoA antibody (Cell Signaling 1:1000). Quantification was calculated as the ratio of total RhoA/loading control: active RhoA/loading control from 4 samples per genotype, run on three separate blots. All controls recommended in the kit were run on each Western blot.

### Explant cultures

Left lung lobes were isolated from E11.5 mice and cultured as described (Dean et al., 2005).

### Organotypic culture and Scrib morpholino knock-down

E12.5 lungs were dissected, removing the primary bronchi and digested in 2 mls 0.3% trypsin, 45 min 37 °C. Lungs were transferred into 2 ml 1:1 DMEM:F12 Invitrogen and 10% FBS, non-essential amino acids, 200 mM glutamine and 50 μg/ml Penicillin–streptomycin (Sigma). Lung tissue was disassociated into a single cell suspension with a 23-gauge needle and centrifuged at 1000 rpm 5 min. Cells were re-suspended, counted and diluted 1:1 in Geltrex (Invitrogen) before adding control (CCTCTTACCTCAGTTACAATTTATA 3′ fluorescein) or Scrib (GAGCGGGATGCACTTCAGCATGATG 3′ fluorescein) morpholinos, MO (Gene Tools) to a final concentration of 10 μM and then plated in a slide chamber (Lab Tek II). After 24 or 48 h cultures were fixed and immunostained.

### Morpholino knock-down of Scrib function and time-lapse movies

Intact E11.5 lungs from transgenic mice expressing GFP from the beta-actin promoter (Hadjantonakis et al., 1998) were cultured as described (Yates et al., 2010). Specific morpholino oligonucleotides against Scrib or control (above, Gene-Tools) were added to the media at final concentration of 15 μM at day zero (Dean et al., 2005). Lung explants were cultured for 48 h and then either fixed for immunofluorescence or used for time-lapse imaging on a Zeiss LSM 510 confocal microscope equipped with a controlled stage incubator. Images were taken every 4 min over a period of 24 h. Acquired time-lapse images were exported using LSM software and saved as Quick Time movies using 20 frames/s.

### Statistical analysis

All statistics and image analyses were computed using Excel, Graphpad, Velocity and Imaris software. Mann–Whitney *U*-test was conducted for image analysis shown in Fig. 4 comparing the orientation of cell migration in lung explants. Error bars represent standard error of mean and significance was scored using unpaired two-tailed *t*-tests.

## Results

### Scrib is required for normal lung development

**Fig. S1:**
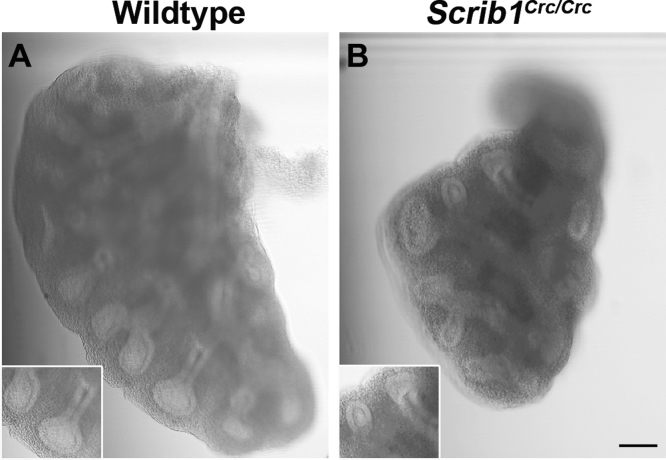
Confocal DIC images of E14.5 wildtype and *Scrib1*^*Crc/Crc*^ left lung lobes. Confocal DIC images of E14.5 wildtype (A) and *Scrib1*^*Crc/Crc*^ (B) left lung lobes. Homozygous mutant lungs are smaller with fewer branches and narrower lumina (see insets for detail).

**Fig. 1:**
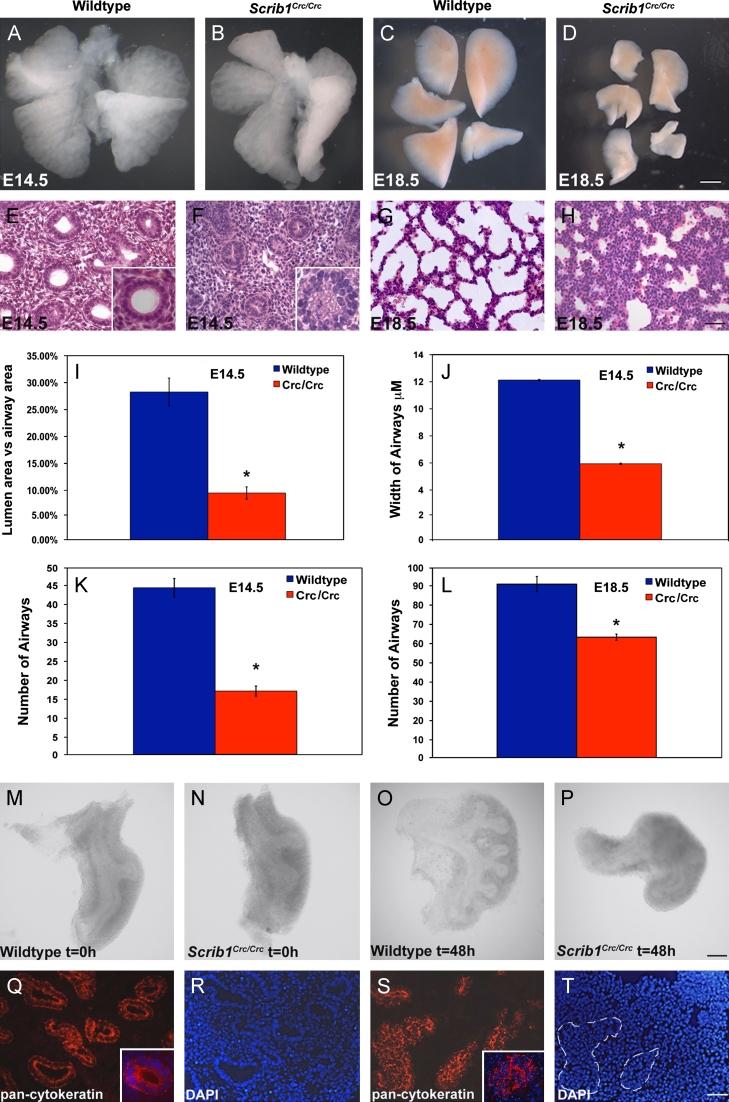
*Scrib1* is required for normal lung development. E14.5 ((A) and (B)) and E18.5 ((C) and (D)) wildtype ((A) and, (C)) and *Scrib1*^*Crc/Crc*^ lungs ((B) and, (D)) show mutant lung lobes are smaller and misshapen. Histological analysis of E14.5H&E stained sections ((E) and (F)) shows fewer, less organised epithelial airways in *Scrib1*^*Crc/Crc*^ lungs ((F), (K): wildtype mean=44.42 μM, ±2.43, *Crc* mean=17.17 μM, ±1.64), with reduced lumen area ((I): wildtype mean=28.2%, ±2.93, *n*=39, *Crc* mean=9.50% ±1.12, *n*=35), compared with wildtype ((E), (I) and (K)). Quantification of airway width highlights the reduced diameter of *Scrib1*^*Crc/Crc*^ lumina ((J): *Crc* mean=5.94 μM, ±0.05 compared to wildtype mean=12.13 μM, ±0.05). By E18.5 severe hypoplasia is evident, with significant reduction in airway number in *Scrib1*^*Crc/Crc*^ lungs ((H), (L): mean=63.33 μM, ±1.61) compared to wildtype ((G), (L): mean=90.89 μM, ±3.97). E11.5 left lung lobes from wildtype (M) and *Scrib1*^*Crc/Crc*^ (N) with identical numbers of buds at *t*=0 were cultured *ex vivo*. After 48 h *Scrib1*^*Crc/Crc*^ lungs had developed far fewer new buds (P) than wildtype lungs (O); representative images for each timepoint are shown. Immunostaining of E14.5 transverse cryosections with anti-pan-cytokeratin ((Q) and (S)) highlights disorganisation of epithelial cells in *Scrib1*^*Crc/Crc*^ lungs (S) compared to controls (Q). Epithelial cells are difficult to distinguish by DAPI staining in *Scrib1*^*Crc/Crc*^ (T) but are easily distinguished in wildtype (R). Scale bars; A–D 62.5 μM, E–H 25 μM, M–P 63 μM, Q–T 25 μM, insets in E, F 5 μM; *P<0.05.

The *Circletail* mutation disrupts the function of mouse Scribble (Murdoch et al., 2003). Lungs from *Scrib*^*Crc/Crc*^ mutant embryos show gross developmental abnormalities (Fig. 1). Murine lungs initiate from the ventral foregut at approximately E9.5 and by E11.5 all five primordial lobes are visible. Gross morphology of *Scrib*^*Crc/Crc*^ lungs appeared unaltered at E11.5 but was markedly disturbed at E14.5 as the lung lobes were misshapen and smaller than controls (Fig. 1A and B, Fig. S1,A and B Supplementary material). At E18.5 the distorted shape and reduced size of *Scrib*^*Crc/Crc*^ lungs was pronounced (Fig. 1D compared to wildtype in C).

Histological analysis showed that lung epithelial and mesenchymal cells were densely packed within the mutant tissue; most notably *Scrib* homozygous lungs contained far fewer visibly open lumina at E14.5 (Fig. 1E and F). Quantification revealed a 33% reduction in lumen area as a percentage of total airway area in *Scrib*^*Crc/Crc*^ (Fig. 1I), and a significant reduction in both the width (Fig. 1J) and total number of airways (Fig. 1K) compared to wildtype littermate lungs. Epithelial cell organization within airways was altered from simple columnar epithelium predominant in wildtype lung sections to multilayered, apparently pseudostratified epithelium in *Scrib* homozygotes. By E18.5, H&E staining revealed reduced septation resulting in fewer, narrower sacculae (Fig. 1G, H and L).

As *Scrib*^*Crc/Crc*^ mutant embryos ultimately have a restricted intrathoracic space, which can impact branching morphogenesis, we performed *ex vivo* culture to compare development of wildtype and *Scrib*^*Crc/Crc*^ lungs without this possible secondary effect. Starting with the same number of terminal buds in wildtype and mutant E11.5 lungs at *t*=0 (Fig. 1M and N), after 48 h culture *Scrib*^*Crc/Crc*^ lungs were smaller with significantly fewer end buds (wildtype mean 8.5, ±0.33, *n*=13; *Crc* mean 2.75,±0.75 *n*=4, *p*=0.0001; Fig. 1O and P). Thus the *Scrib* mutation in *Crc* alters lung morphology resulting in fewer epithelial airways with absent or narrower diameter lumina and later on, to fewer, narrower sacculae. We were unable to examine postnatal lung function in *Scrib*^Crc/Crc^ mice as they die at birth from neural tube defects, though it is likely that the defects observed would severely impact lung function.

### Disruption of *Scrib* perturbs epithelial tube formation

To explore the cellular basis for the *Scrib*^*Crc/Crc*^ lung defects, we analyzed the expression of markers of lung epithelial differentiation and determined whether cell proliferation or cell death was altered. Immunostaining of E14.5 lung sections with pan-cytokeratin to visualize epithelial airways and DAPI to detect the nuclei showed in wildtype lungs that epithelial cells were easily distinguished from mesenchyme and that the simple columnar epithelial cells were regularly arranged around a distinct central lumen (Fig. 1Q and R). In contrast, *Scrib*^*Crc/Crc*^ epithelial cells were not properly aligned with respect to each other and in the majority of cases a lumen was barely visible (Fig. 1S and T), indicating severe defects in epithelial tube structure, consistent with data above showing that many *Scrib*^*Crc/Crc*^ airways have narrow lumina and the epithelial cells appear pseudostratified.

**Fig. S2:**
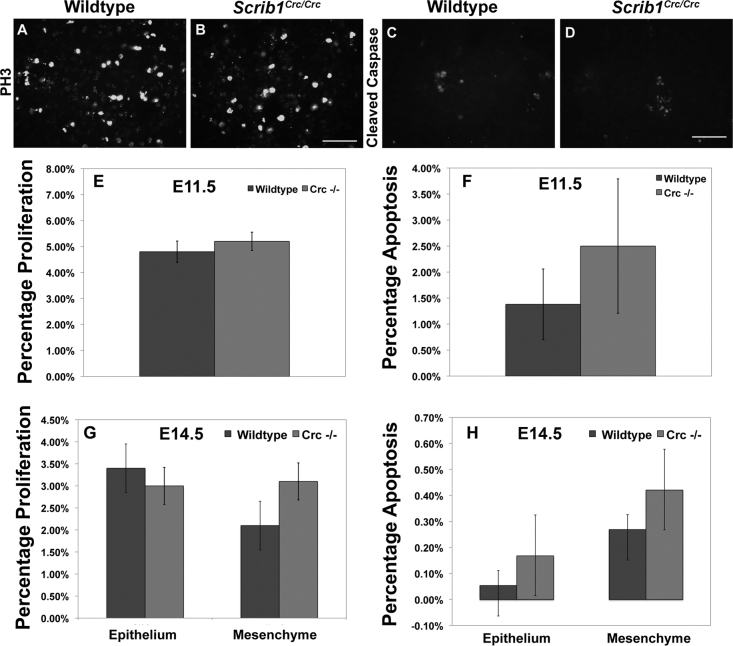
Proliferation and apoptosis are unaffected in *Scrib1*^*Crc/Crc*^ lungs. E14.5 wildtype ((A) and (C)) and *Scrib1*^*Crc/Crc*^ ((B) and (D)) sections immunostained with anti-phosphohistone-3 (PH3) ((A) and (B)) to assess proliferation or anti-cleaved caspase-3 ((C) and (D)) to assess apoptosis. Quantification at both E11.5 ((E) and (F)) and E14.5 ((G) and (H)) shows no difference in the percentage of proliferating cells ((E) and (G)) or the percentage of cells undergoing programmed cell death ((F) and (H)) between *Scrib1*^*Crc/Crc*^ and wildtype littermates. (E) wildtype *mean*=4.8%±0.38, *n*=9, *Crc mean*=5.2%±0.34, *n*=9. (F), wildtype *mean*=1.38%, ±0.63, *n*=8, Crc *mean*=2.50%±1.18, *n*=6. (G), Wildtype epithelium mean=3.40%, ±0.54, *n*=16, Crc epithelium mean=3.04%, ±0.41, *n*=21; wildtype mesenchyme mean=2.11%, ±0.28, *n*=8, Crc mesenchyme mean=3.05%, ±0.32, *n*=14. (H), wildtype epithelium mean=0.05%, ±0.05, *n*=8, Crc epithelium mean=0.17%, ±0.10, *n*=6; wildtype mesenchyme mean=2.71%, ±0.15, *n*=7, Crc mesenchyme mean=0.42%, ±0.16, *n*=6 Scale bars; A–D 12.5 μM.

As *Scrib*^*Crc/Crc*^ lungs were smaller than controls, we examined cell proliferation and apoptosis at both E11.5 and E14.5. No significant differences relative to wildtype were detected at either time-point in the percentage of cells positive for phospho-histone H3 (PH3) (Fig. S2A, B, E and G) or cleaved caspase 3 (Fig. S2C, D, F and H), indicating the reduced size of *Scrib*^*Crc/Crc*^ lungs is not due to changes in proliferation or apoptosis. Calculation of the mean number of nuclei per airway area, however, revealed that cell density was significantly increased in mutant lungs from 0.10±0.003 (*n*=33) in wildtype, to 0.14±0.004 (*n*=34) in *Scrib*^*Crc/Crc*^ (*p*=<0.001). This suggests that disrupted cellular organisation and a loss of lumen/air space, may account for the reduction in lung size.

**Fig. S3:**
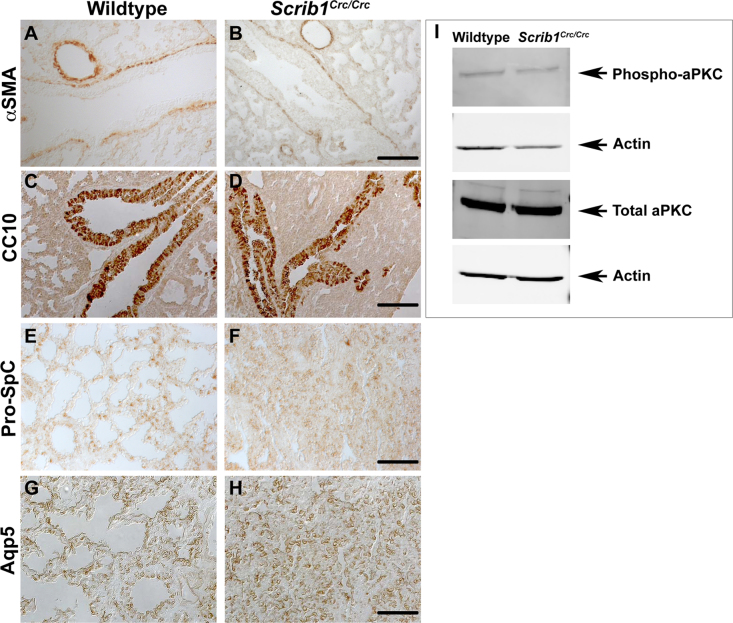
Differentiation is unaffected in *Scrib1*^*Crc/Crc*^ lungs. Analysis of two proximal airway markers, αSMA ((A) and (B)) and Clara cell-10 ((C) and (D)), and two distal airway markers, Pro-SpC ((E) and(F)) and Aqp5 ((G) and (H)), by immunohistochemistry revealed no obvious differences in differentiation of *Scrib1*^*Crc/Crc*^ lungs ((B), (D), (F) and (H)) compared to wildtype ((A), (C), (E) and (G)). Western blotting revealed the levels of both total and phospho-aPKC remained similar in *Scrib1*^*Crc/Crc*^ lungs compared to wildtype littermates (I). Scale bars; A–H 12.5 μM.

To assess whether cellular differentiation was affected, E18.5 lung sections were immunostained with α-smooth muscle actin (*α*–SMA, proximal airway marker), the Clara cell marker, CC-10, pro-Surfactant protein C (pro SP-C, a marker of distal type II alveolar cells) and aquaporin-5 (type I alveolar cells (Fig. S3 Supplementary material). No differences in expression of these markers were observed between *Scrib*^*Crc/Crc*^ and wildtype littermates. Thus it appears that the morphological changes evident in *Scrib*^*Crc/Crc*^ lungs do not result from altered proliferation, apoptosis or differentiation but instead result from a disruption in epithelial organization, leading to reduced air space volume.

### *Scrib*^*Crc/Crc*^ lung epithelia display cytoskeletal defects but A/B polarity is not affected

Our previous studies showed that *Celsr1* and *Vangl2* proteins signal via the PCP pathway to regulate cytoskeleton dynamics that are necessary for lung branching morphogenesis (Yates et al., 2010). Since Scrib can effect both PCP and A/B polarity (Bilder and Perrimon, 2000; Courbard et al., 2009; Montcouquiol et al., 2003; Zhan et al., 2008), we wished to ascertain the relevant pathway(s) through which Scrib was acting to regulate lung development.

**Fig. 2:**
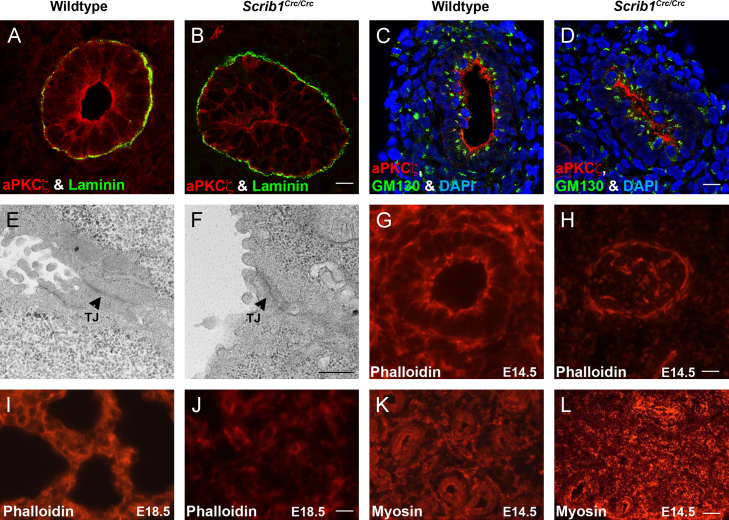
Apical–basal polarity appears unaffected but the cytoskeleton is disrupted in *Scrib1*^*Crc/Crc*^ lungs. Immunostaining of E11.5 transverse lung cryosections revealed no obvious defects in A/B polarity in *Scrib1*^*Crc/Crc*^ airways. aPKCς, is present at the apical surface in both *Scrib1*^*Crc/Crc*^ (red (B) and (D)) and wildtype sections (red (A) and (C)). Laminin localises to the basal side of the airways in both wildtype and mutant (green (A) and (B)). The Golgi marker GM130 (green (C) and (D)) is present on the apical side of DAPI-stained (blue (C) and (D)) nuclei in both *Scrib1*^*Crc/Crc*^ (D) and wildtype (C) lung sections. T.E.M. analysis of E16.5 lung revealed no apparent disruption to A/B polarity; in *Scrib1*^*Crc/Crc*^ (F) and wildtype controls (E), airway epithelial cells were orientated with their apical surface (recognised by the presence of microvilli) towards the lumen and apparently intact tight junctions (arrowheads). (TJ), tight junction. E14.5 ((G) and (H)) and E18.5 ((I) and (J)) Phalloidin-stained cryosections reveal severe disruption to the actin cytoskeleton in *Scrib1*^*Crc/Crc*^ ((H) and (J)) lungs; cortical actin was discontinuous and frequently not visible. In wildtype littermates, cortical actin was visible around the cell membranes (G, I). Immunostaining for non-muscle myosin IIA (K, L) also revealed disrupted distribution in *Scrib1*^*Crc/Crc*^ (L) lungs compared to wildtype (K). A–D 125 μM plus ×3 zoom, E–F 5 μM, G–J 5 μM K and L 25 μM.

We carefully assessed whether the *Circletail* mutation leads to disrupted A/B polarity in E14.5 transverse lung sections. Immunostaining for the apical membrane marker aPKCζ and the basement membrane component Laminin revealed no major disruption to A/B polarity (Fig. 2B compared to wildtype in Fig. 2A). This was confirmed by triple-labelling with aPKCζ, DAPI and the Golgi marker GM-130 (Fig. 2C and D). Western blotting revealed no difference in levels of either total or phospho- aPKC between wildtype and *Scrib*^*Crc/Crc*^ lungs, indicating that the activity of this apical protein was unaffected (Fig. S3 Supplementary material). In addition, there was no evidence of aberrant A/B polarity by TEM analysis of wildtype and *Scrib*^*Crc/Crc*^ lung epithelium (Fig. 2E and F).

The end result of activity of the PCP pathway is modification of the cytoskeleton across groups of cells, usually epithelia, to direct coordinated cell movement/organization. To examine the cytoskeleton, lung sections were stained with phalloidin to reveal the filamentous F-actin network. At E14.5, F-actin enrichment normally observed in both the apical and basolateral membranes of wildtype airway epithelial cells (Fig. 2G) was not visible in *Scrib*^*Crc/Crc*^ lungs (Fig. 2G and H). Rather than a continuous band of cortical actin highlighting the sub-apical surface, the F-actin in mutant airways was observed in multiple discontinuous patches. At E18.5, phalloidin staining showed further disruption to the F-actin network in *Scrib*^*Crc/Crc*^ lungs (Fig. 2I and J). Notably, the fluorescence intensity of phalloidin was lower in the homozygous epithelium and mesenchyme, suggesting a reduction in polymerised actin. Distribution of another critical cytoskeletal protein, non-muscle myosin IIA, was also perturbed in *Scrib*^*Crc/Crc*^ (Fig. 2L) compared to control (Fig. 2K). Thus, *Scrib* mutant lungs show a severe perturbation in cytoskeletal organisation.

### Scrib modulates localisation of the core PCP proteins Celsr1 and Vangl2 in lung epithelium and genetically interacts with *Vangl2*

**Fig. 3:**
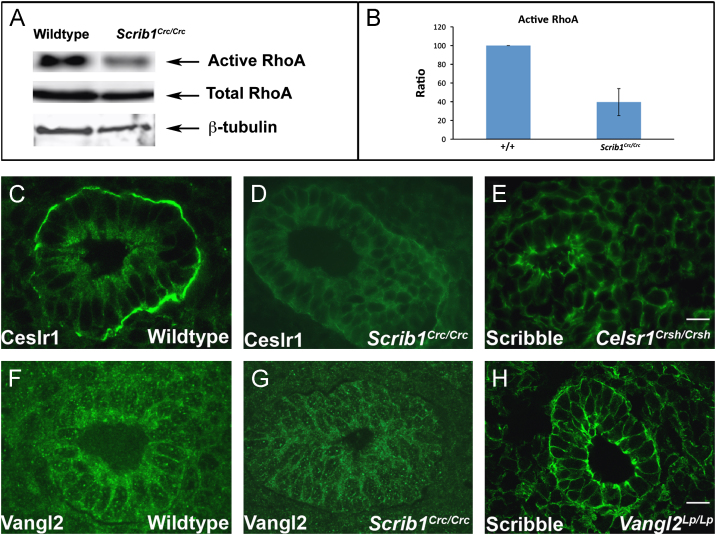
Active RhoA is reduced in *Scrib1*^*Crc/Crc*^ lungs and Scrib1 is required for correct localisation of Celsr1 and Vangl2 in lung epithelium. Western blotting (A) reveals a 60% reduction in the relative levels of active RhoA in E13.5 *Scrib1*^*Crc/Crc*^ lungs compared to wildtype littermates following active Rho pull-down and detection with RhoA (21KDa) antibody; (B) relative levels in wildtype: 100; *Scrib1*^*Crc/Crc*^: 39.6, *p*=0.01, *n*=4 for each genotype. No alteration in levels of total RhoA was observed between *Scrib1*^*Crc/Crc*^ and wildtype (A). Levels of total and RhoA GTP were normalised to loading control levels detected with β-tubulin (50 kDa). E14.5 wildtype transverse lung cryosections immunostained with anti-Celsr1 shows localisation to epithelial cell membranes, at the apical and particularly around the basal side of airways in the basement membrane (C). In *Scrib1*^*Crc/Crc*^ sections, the basement membrane localisation is lost (D). In the *Celsr1*^*Crsh/Crsh*^ mutant, Scrib1 is weaker around epithelial membranes and appears more diffuse (E, compare to Scrib1 localization in wildtype lung I). At E14.5 Scrib1 is localised to the plasma membrane of epithelial and mesenchymal cells and is highly enriched towards the apical surface of airways (I). Scrib1 is absent in E14.5 *Scrib1*^*Crc/Crc*^ lungs (J). Punctate Vangl2 immunostaining is enriched towards the apical surface in wildtype sections (F) but this enrichment is less prominent in *Scrib1*^*Crc/Crc*^ (G). In *Vangl2*^*Lp/Lp*^ airways, Scrib1 immunostaining is unaffected (H). Scale bars; C–G, J 125 μM plus ×3 zoom, H 125 μM plus 2.5 zoom, I 125 μM plus 2 zoom.

The relatively normal A/B polarity but disrupted cytoskeleton suggested that Scrib might operate through the PCP signalling pathway in mammalian embryonic lungs. Consistent with this hypothesis, detection of activated RhoA (Rho GTP) by pull-down assay revealed a 60% reduction in levels of Rho GTP, a downstream effector of the PCP pathway, in *Scrib*^*Crc/Crc*^ mutant *versus* wildtype lungs (Fig. 3A, B and Fig. S6 supplemental material).

**Fig. S4:**
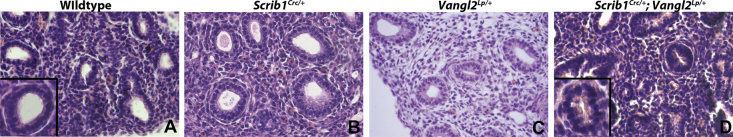
*Scrib1* shows genetic interaction with *Vangl2*. E14.5 paraffin sections from wildtype littermates (A *n*=3), *Scrib1*^*Crc/+*^ (B *n*=2), *Vangl2*^*Lp/+*^ (C *n*=3) or *Scrib1*^*Crc/+*^; *Vangl2*^*Lp/+*^ double heterozygotes (D *n*=4) were stained with H&E to compare histology. Airways in *Scrib1*^*Cr//+*^(B) and *Vangl2*^*Lp/+*^ (C) heterozygous lung sections show open organised airways, similar to those seen in wildtype sections (A) although minor epithelial disorganisation and slight narrowing of lumina is observed in *Vangl2*^*Lp/+*^. In contrast, the lungs of compound heterozygotes (D) contain disorganised airways with severely reduced or absent lumina. Insets in A and D show a single epithelial airway at high magnification. Scale bars; A and B 25 μM, insets 12.5 μM.

To look for additional evidence that Scrib acts within the PCP pathway in lung, we examined whether Scrib modulates the localisation of either Celsr1 or Vangl2 proteins. In E14.5 *Scrib*^*Crc*/Crc^ lungs, Celsr1 localization was markedly altered, being redistributed from the polarised, membrane-bound basal location observed in wildtype (Fig. 3C) to a more diffuse distribution around the periphery of epithelial cells as well as in the cytoplasm (Fig. 3D). Staining in the basement membrane was markedly diminished, although the basement membrane was present in *Scrib*^*Crc/Crc*^ lung epithelia, and is detected by anti-laminin immunostaining (see Fig. 2B). Vangl2 enrichment towards the apical side of wildtype airways (Fig. 3F) was not observed in *Scrib*^*Crc/Crc*^ (Fig. 3G), indicating a subtle change in spatial localisation of Vangl2, in agreement with previous studies (Phillips et al., 2007). In contrast, Scrib protein appeared largely unaltered in *Celsr1*^*Crsh/Crsh*^ and *Vangl2*^*Lp/Lp*^ mutant lungs (Fig. 3E and H) relative to wildtype (Fig. 3I). *Scrib*^*Crc/Crc*^ lung tissue immunostained with anti-Scrib almost completely lacked Scrib expression, indicating both specificity of the antibody and that Scrib protein is essentially absent in *Circletail* lung (Fig. 3J). Thus, in *Scrib* mutants the asymmetric localization of both Celsr1 and Vangl2 is altered, providing further evidence that Scrib affects the PCP pathway. As further evidence that Scrib acts via the PCP pathway in lung development we intercrossed *Scrib*^*Crc/+*^ and *Vangl2*^*Lp/+*^ mice (Fig. S4 Supplemental material) and compared the embryonic lungs to wildtype littermate controls (Fig. S4A) and to both *Scrib*^*Crc/+*^ (Fig. S4B) and *Vangl2*^*Lp/+*^ (Fig. S4C) heterozygotes. Lungs from these compound heterozygotes (Fig. S4B) displaying the open neural tube defect, craniorachischisis, also showed lung defects. The airways of compound heterozygotes displayed a severe phenotype with narrow or absent lumina compared to either wildtype littermates or to *Scrib*^*Crc/+*^ or *Vangl2*^*Lp/+*^ heterozygotes; although we did observe mild defects in the *Vangl2*^*Lp/+*^ airways that were distinct from either wildtype or *Scrib*^*Crc/+*^ heterozygotes. The epithelial disorganisation and lung lumen defects present in *Scrib*^*Crc/+*^*; Vangl2*^*Lp/+*^ lungs phenocopied those of *Scrib*^*Crc/Crc*^ homozygotes (Fig. 1F) thereby indicating a genetic interaction.

### Real-time imaging reveals uncoordinated shifting between lung epithelial cells in the presence of Scrib morpholinos

**Fig. S5:**
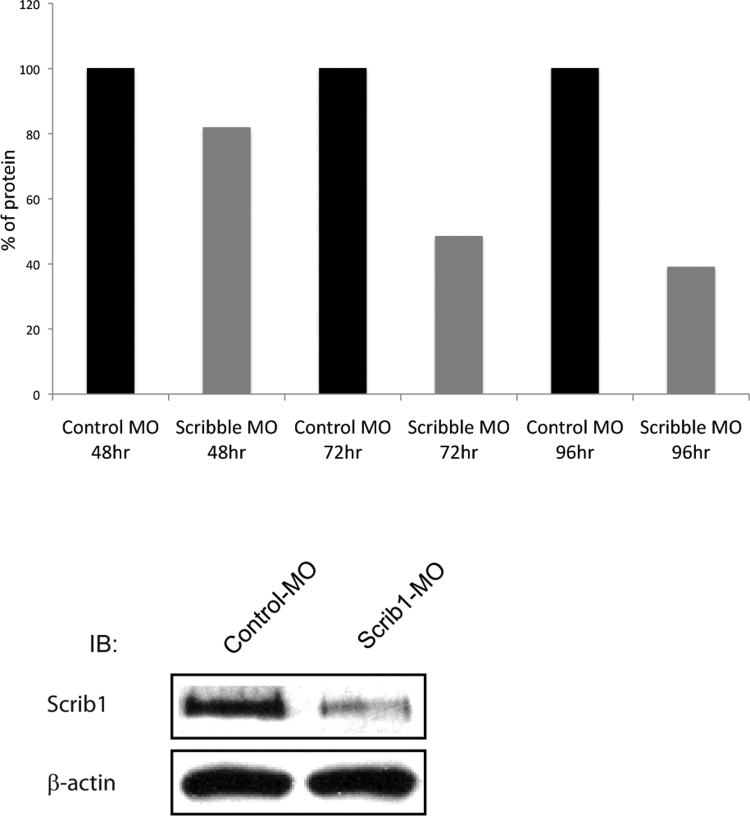
Validation of morpholino knockdown. E11.5 lungs were cultured for between 48 and 72 or 96 h in the presence of *Scrib1* or control morpholinos (MO) and then protein extracted for Western blotting with anti-Scrib1 antibodies; *n*=3 explants (minimum) for each condition at each timepoint. Quantification of relative protein levels following Western blotting revealed an increasing reduction in Scrib1 protein following *Scrib1* morpholino treatment over time. IB, immunoblotting shows an example of data from 96 h culture.

**Fig. S6:**
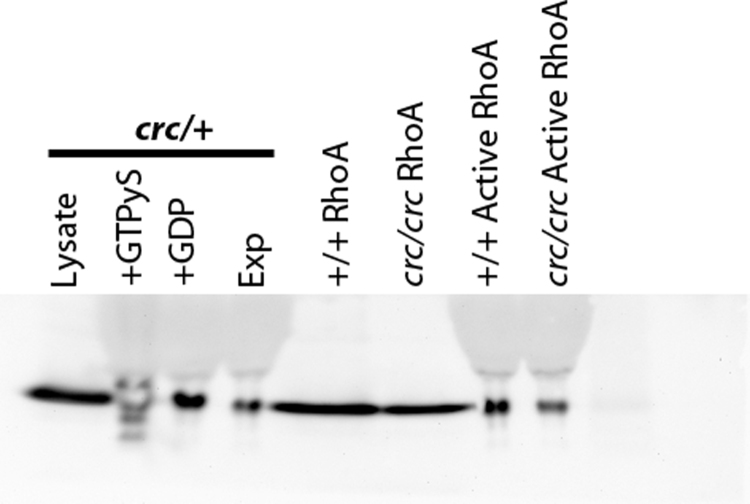
Western blot showing comparison of total RhoA and active RhoA in wilddtype and *Scrib1*^*Crc/Crc*^. Raw data of image shown in Fig. 3A.

Static images of lung development cannot reveal the dynamic behaviour of cells as they undergo lung morphogenesis. To gain insight into how Scrib regulates epithelial tube morphology we conducted time-lapse video microscopy of wildtype lung explants treated either with control or *Scrib* morpholinos (MO). Knock-down efficiency was validated by Western blotting to determine the level of Scrib protein in explants after 48, 72 and 96 h in culture (Fig. S5 Supplementary material).

**Fig. 4:**
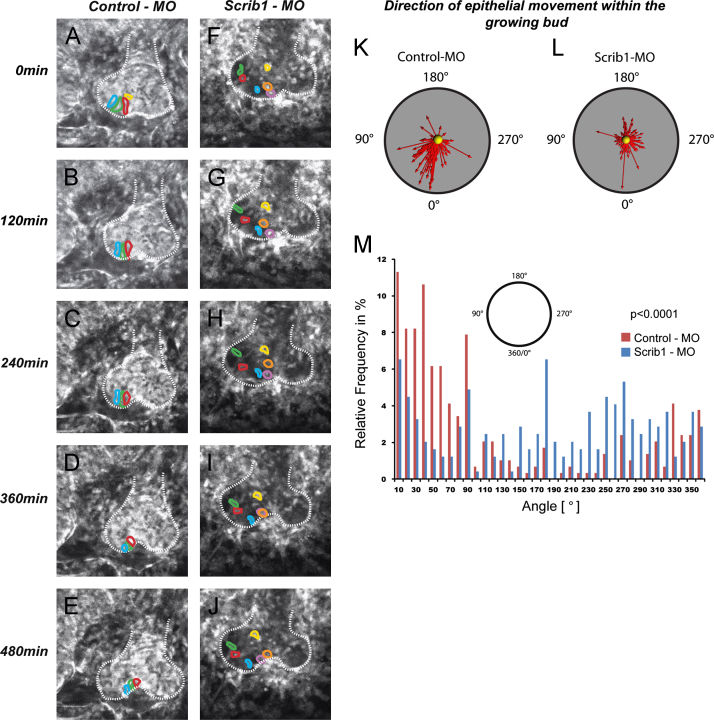
Morpholino knock-down of Scrib1 leads to misalignment of epithelial cells in *ex vivo* lung culture. E11.5 lung explants from β-actin promoter driven GFP embryos were cultured for 48 h with control or *Scrib1* morpholinos and subsequently imaged over a period of 24 h (see Movies 1 and 2). During this 24 h time period, Scrib protein levels reduce from 80% of wildtype to 50%. Five time points from this series are depicted. Selected cells from control (A–E) or *Scrib1* MO (F–J) treated explants were highlighted with different colours and their movements tracked over time. Epithelial cells from control MO treatment maintain a close neighbor–neighbor relationship, whereas with *Scrib1* MO treatment, the cells are more loosely associated and often change their position (in particular, compare the relationship between the cells marked in red and green and in purple and blue and in purple and orange). The distribution of the direction of epithelial cell movements revealed by tracking ∼150 cells within the distal epithelium shows a close to random pattern in *Scrib1*-MO treated lung explants (L) compared to control (K) which shows more directional cell movements. Graphical representation showing the frequency with which cells migrate at each angle (M). *P*<0.0001, Mann–Whitney *U* test.

Culture of E11.5 wildtype lungs with *Scrib* MO resulted in a striking reduction in epithelial cohesion. We observed considerable shifting of individual distal epithelial cells relative to one another, leading to changes in relative positioning of neighbouring cells within forming buds (Fig. 4 and Movie 2, Supplementary material). In control MO lung explants, the epithelial cells maintained their position with respect to their neighbours (Fig. 4 and Movie 1, Supplementary material). Moreover, the direction of epithelial cell movements within the distal epithelium showed a random pattern in *Scrib* MO treated lung explants relative to the directional cell movements in control MO treated (Fig. 4K, L and M). Thus, upon loss of Scrib function, alignment and close association of epithelial cells were disrupted, suggesting that Scrib functions to regulate epithelial contacts and thereby maintain epithelial integrity and the luminal space.

The following is the Supplementary material related to this article Video 1, Video 2

Video 1 Time-lapse movies of control MO treated β-actin driven GFP lung explants. After 48 h of culture, lungs were transferred to a Zeiss LSM 510 confocal microscope equipped with controlled stage incubator. Images were taken every 4 min over a 24 h period. Acquired time-lapse series were exported using LSM software and saved as Quick Time movies using 20 frames/s.

Video 2 Time-lapse movies of Scribble MO treated β-actin driven GFP lung explants. After 48 h of culture, lungs were transferred to a Zeiss LSM 510 confocal microscope and imaged as described for Movie 1.

### Scrib is localised to the cell membranes and associates with junctional proteins

**Fig. 5:**
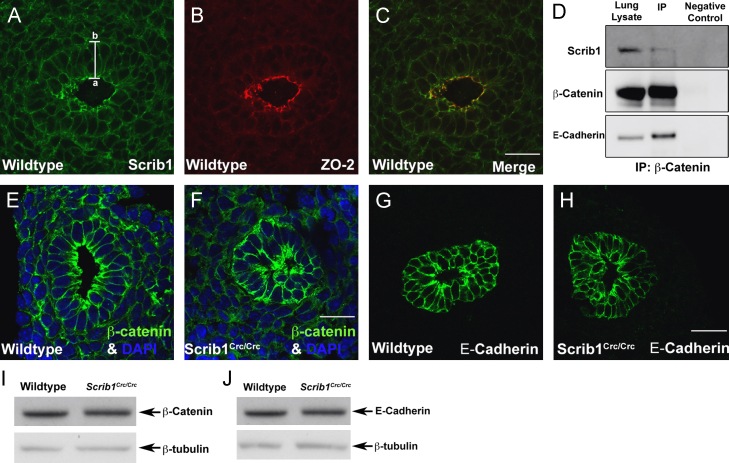
Scrib1 is localised to the cell membrane and tight junctions and interacts with β-catenin. Transverse cryosection of E11.5 ((A)–(C)) wildtype lung immunostained for Scrib1 ((A) and (C)) shows Scrib1 is localised to membranes of epithelial and mesenchymal cells; strong staining is also observed apically, where Scrib1 co-localises with ZO-2 ((B) and (C)). Immunoprecipitation with β-Catenin antibody revealed an interaction with Scrib1 in endogenous lung tissue, suggesting these proteins physically interact in the lung (D). A thin band of β-catenin is observed around the basolateral membranes of E14.5 wildtype airways and is predominant at the apical membrane (E). In *Scrib1*^*Crc/Crc*^ airways, β-catenin appears diffuse and unevenly distributed around cells (F). E-cadherin distribution is largely unaltered in *Scrib1*^*Crc/Crc*^ airways (G) compared to controls (H). Western blotting shows no significant difference in the quantity of β-catenin (95 kDa) (I) or E-cadherin (120 kDa) protein (J) between E14.5 wildtype and *Scrib1*^*Crc/Crc*^ whole lung. Scale bars; A–C 125 μM plus ×2 zoom, E, F, G and H 125 μM plus ×3 zoom a, apical, b, basal.

To explore further the mechanism of Scrib function, we first determined the spatial localisation of Scrib in the developing lung. Immunostaining of E11.5 transverse wildtype lung sections showed strongly positive staining towards the apical surface of airway epithelial cells and this co-localized with the tight junction (TJ) marker ZO-2 (Fig. 5A and C). Apical localisation of Scrib is consistent with previous reports in other cells and tissues (Bilder and Perrimon, 2000; Ivanov et al., 2010a; Metais et al., 2005). In addition, lower levels of Scrib were observed around the entire plasma membrane of epithelial and mesenchymal cells (Fig. 5A). To determine which junctional components Scrib physically interacts with in the developing lung we conducted a series of immunoprecipitation experiments using E13.5/14.5 mouse lung lysates. We found a specific interaction between β-catenin and Scrib, indicating that these two proteins physically interact in endogenous lung tissue (Fig. 5D). In contrast we were unable to detect an interaction between ZO-2 and Scrib following separate immunoprecipitation experiments with antibodies to either of these proteins.

### Scrib contributes to adherens and tight junction integrity

The interaction of Scrib with β-catenin and its co-localisation with ZO-2 suggested that Scrib contributes to adherens and/or tight junction integrity in lung epithelial cells. We therefore investigated whether adherens and tight junction proteins were normally localised in homozygous mutant airways. E14.5 transverse lung cryosections immunostained for β-catenin showed a thin band of β-catenin around the entire basolateral membrane of wildtype airway epithelial cells, with particularly strong immunostaining towards the apical surface, where the lumen is expanding (Fig. 5E). In *Scrib*^*Crc/Crc*^ airways, β-catenin distribution was markedly altered and variable. Thick bands of protein were localised around the basal and lateral sides of many airway cells, whereas in other cells, localisation to lateral membranes was almost absent or restricted to a portion of the lateral membranes only (Fig. 5F). In contrast, E-cadherin localisation appeared largely unaltered in *Scrib*^*Crc/Crc*^ *versus* wildtype airways, although we occasionally noted some subtle but variable changes in E-cadherin such as uneven distribution around the basolateral membranes (Fig. 5G and H). Quantification of β-catenin and E-cadherin by Western blot revealed no change in overall levels of these proteins in *Scrib*^*Crc/Crc*^ compared to wildtype lungs (Fig. 5I and J).

**Fig. 6:**
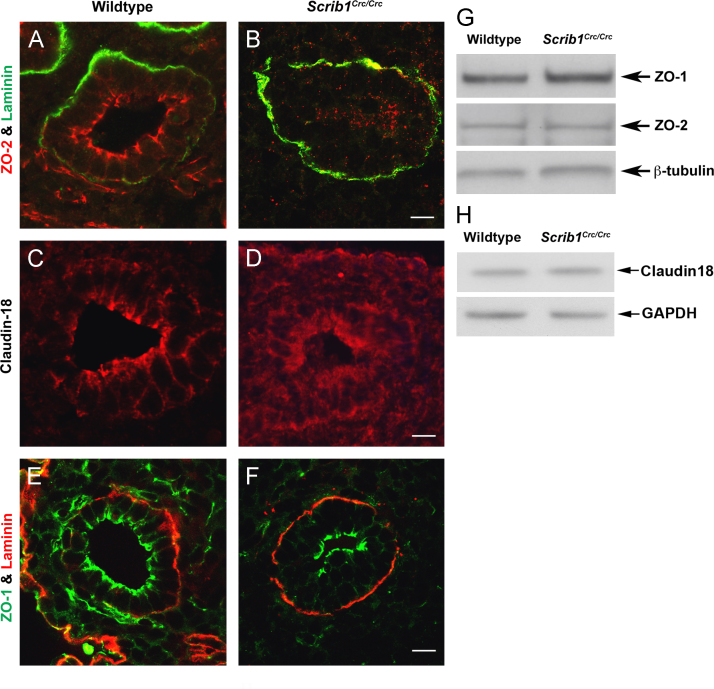
Specific tight junction proteins are disrupted in *Scrib1*^*Crc/Crc*^ lungs. E14.5 transverse wildtype ((A), (C) and (E)) and *Scrib1*^*Crc/Crc*^ ((B), (D) and (F)) lung sections immunostained for tight junction markers. Apical localisation of ZO-2 is maintained in *Scrib1*^*Crc/Crc*^ lungs (B), but its localisation is abnormal compared to wildtype (A). Claudin-18 is also mislocalised in *Scrib1*^*Crc/Crc*^ epithelial airways (D) compared to wildtype (C). ZO-1 appears normal in *Scrib1*^*Crc/Crc*^ lungs ((E) and (F)). ((G) and (H)) Levels of ZO-1 (220 kDa), ZO-2 (160 kDa) (G) and Claudin-18 (23 kDa) (H) were similar in wildtype and *Scrib1*^*Crc/Crc*^ lungs by Western blot. Scale bars; A–F 125 μM plus ×3 zoom.

Because the stability of tight junctions is strongly linked to that of adherens junctions and we had previously observed co-localisation of Scrib and the tight junction protein ZO-2 (Fig. 5C), this prompted us to examine the distribution of ZO-2 and additional TJ proteins, ZO-1 and Claudin-18 in *Scrib*^*Crc/Crc*^ lung tissue. The sub-cellular distribution of ZO-2 was severely perturbed in *Scrib*^*Crc/Crc*^ lung epithelia, although Western blotting revealed no difference in the overall level of ZO-2 between wildtype and mutant lung (Fig. 6A, B and G). The distribution, but not the overall levels, of Claudin-18 was also markedly perturbed in *Scrib*^*Crc/Crc*^ lungs. Claudin-18 staining appears more diffuse throughout the mutant epithelium and mesenchyme rather than being predominantly restricted towards the apical surface of wildtype epithelial cells where the tight junctions are located (Fig. 6C, D and H). Interestingly, we did not detect disruption to another zonula occludens protein, ZO-1, in *Scrib*^*Crc/Crc*^ lungs (Fig. 6E, F and G), suggesting that the interaction between Scrib and TJ proteins is selective.

Intact adherens and tight junctions are necessary to maintain proper epithelial cell–cell contacts (Gooding et al., 2004; Shin et al., 2006). Our observations that Scrib is localised to both the cell membranes and tight junctions, along with disruption of some tight and adherens junction proteins in *Scrib*^*Crc/Crc*^ epithelial airways, supports the hypothesis that Scrib plays a role in maintaining correct cell–cell adhesion. Our data suggest that in *Crc* mice, disruption of key junctional proteins destabilises airway epithelial cells so that they are unable to maintain their proper alignment and this lack of epithelial cohesion leads to a collapse of the epithelial cells into the lumen and a reduction in luminal area.

### Organotypic cultures reveal a role for Scrib in formation and correct organisation of epithelial cysts

**Fig. 7:**
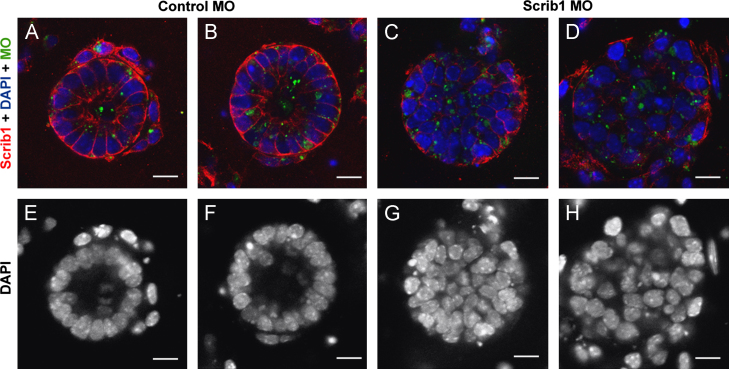
Scrib1 knockdown prevents normal epithelial cyst formation in organotypic cultures. Morpholino (MO, A–D, green) knockdown of Scrib1 ((C) and (D)) in organotypic cultures inhibits re-association of epithelial cells into organized cysts with a centrally located lumen. Scrib1 ((A)–(D), red) and DAPI ((A)–(H), blue/white) staining reveal organised epithelial cells surrounding a centrally located lumen in cultures containing control MO ((A), (B), (E) and (F)). In the presence of *Scrib1* MO, cysts are comprised of misaligned epithelial cells and either a small non-central lumen or, frequently no visible lumen ((C), (D), (G) and (H)). Scale bars; A–H 125 μM ×2 zoom.

Our data indicate that Scrib plays a role in the maintenance of junctional complexes that are necessary for proper epithelial organisation. To determine whether Scrib is required for the formation of *de novo* epithelial cell–cell contacts, we performed organotypic cultures using single cell suspensions of mixed epithelial and mesenchyme cells from E12.5 wildtype mouse lung in the presence of either control or *Scrib* MO. After 48 h culture with control MO, well-ordered cysts had formed, each of which contained a simple columnar epithelium surrounding a visible centrally located lumen (Fig. 7A, B, E and F). In contrast, cells cultured with *Scrib* MO formed cysts containing disorganized epithelial cells and lacked a visible lumen or had a very narrow diameter lumen (Fig. 7C, D, G and H). Knock-down was confirmed by immunostaining for Scrib in control MO-treated (Fig. 7A and B) and *Scrib* MO-treated (Fig. 7C and D) organotypic cultures. These findings are consistent with our *in vivo* results showing that *Scrib*^*Crc/Crc*^ lungs contain fewer epithelial branches with a disorganized epithelium and narrow or absent lumina.

**Fig. 8:**
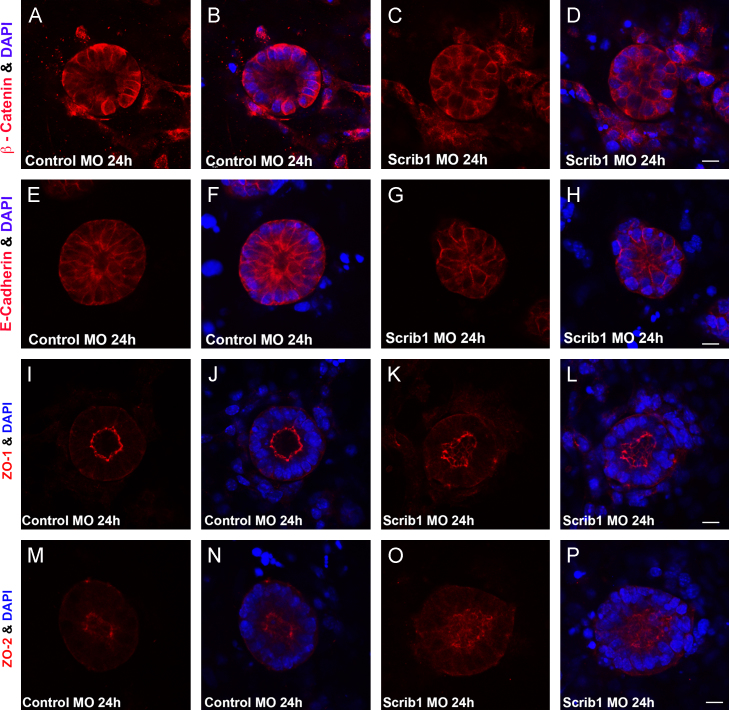
Scrib1 knockdown in organotypic cultures disrupts β-catenin and ZO-2 localization. Morpholino (MO) knockdown of Scrib1 ((C),(D), (G), (H), (K), (L), (O) and (P)) in organotypic cultures compared with control MO ((A), (B), (E), (F), (I), (J), (M) and (N)), followed by immunostaining for junctional proteins revealed selective disruption to key junctional proteins,. β-catenin ((A)–(D) red) and ZO-2 ((M)–(P) red) sub-cellular localisation was significantly disrupted upon Scrib1 knockdown ((C), (D) and (O), (P) compared with (A), (B) and (M), (N)). E-cadherin ((E)–(H) red) did not appear to be visibly altered (compare (G) and (H) with control MO (E) and (F)). ZO-1 ((I)–(L) red) localisation in Scrib1 MO cultures ((K) and (L)) appeared mildly disrupted compared with control MO ((I) and (J)). DAPI staining highlights nuclei ((B), (D), (F), (H), (J), (L), (N) and (P) blue). Scale bars; A–P 5 μM plus 6*x* zoom.

In addition we compared the distribution of junctional proteins in control and Scrib MO-treated organotypic cultures. β-catenin (Fig. 8A–D) and ZO-2 (Fig. 8M–P) displayed significant changes in sub-cellular localisation upon Scrib knockdown whilst E-cadherin (Fig. 8E–H) did not appear to be visibly altered. ZO-1 (Fig. 8I–L) localisation also appeared to be disrupted though the changes were significantly milder than seen for β-catenin or ZO-2. These organotypic culture results are in agreement with our *in vivo* data and indicate that Scrib loss perturbs the localization of some junctional proteins and disrupts cell–cell contacts necessary to maintain epithelial integrity.

## Discussion

### Scrib is required for normal lung branching morphogenesis

Our investigation reveals that Scrib is required for normal lung branching morphogenesis.

The *Scrib* loss-of-function mutation in *Circletail* mice leads to smaller and abnormally shaped lung lobes. Lung epithelial branches show abnormal distribution of the actin–myosin cytoskeleton and considerable cellular disorganisation; the number and width of airways is reduced and they often lack a visible lumen or contain only a narrow diameter lumen. The finding that Scrib is required to maintain proper lumen diameter *in utero* provides novel mechanistic insight into the regulation of lumen morphogenesis in the lung. To date, investigations into how lung lumen diameter is regulated have focused on the roles of foetal breathing movements and on the establishment of ion channels, which facilitate the regulation of lumen size via pumping of fluid into the luminal space (Inanlou et al., 2005; Wilson et al., 2007). The studies presented in this manuscript show that perturbation of Scrib leads to loss of normal cell–cell interactions between airway epithelial cells and that this in turn results in abnormal lumen diameter. Mechanistically, through analysis of mutant lungs, the use of lung explant cultures to dynamically observe lung branching and of organotypic cultures to study lumen organization, we show that Scrib regulates epithelial cell contacts and the sub-cellular distribution of adherens and tight junction proteins, most likely through the association of Scrib with β-catenin in the embryonic lung. Finally, Scrib is required for cytoskeletal re-modelling in the developing lungs, which determines the structure of the epithelial tubes, and is also required for the proper distribution of the PCP proteins Celsr1 and Vangl2, mutations of which also cause defects in lung morphogenesis.

### *Scrib* signals via the PCP pathway to regulate lung development

Scrib is able to signal via the A/B and planar polarity pathways, though the pathways utilised appear largely species-specific. Most *Drosophila* studies show that Scrib is involved in A/B polarity, whilst in mammals Scrib appears to be required for planar polarity. However, a recent *Drosophila* study examined the effect of a *Scrib* hypomorph, in which PDZ domains 3 and 4 are absent (Courbard et al., 2009). This hypomorph exhibits PCP defects whilst A/B polarity is unaffected, indicating that Scrib does contribute to planar polarity in *Drosophila*. It may be that in the complete absence of Scrib, A/B polarity defects are the predominant phenotype and this masks a role in planar polarity. Interestingly, Laprise et al. (2010) have shown that Scrib also affects tube size during *Drosophila* tracheal development, however unlike in mouse, Scrib mutation in *Drosophila* leads to longer tracheal tubes due to a broadening of apical membrane size.

The results presented here indicate that, in the mammalian embryonic lung, *Scrib* operates via the PCP signalling pathway to modulate epithelial tube anatomy, in part via Rho kinase-mediated organisation of the cytoskeleton. In normal lungs, cytoskeletal re-modelling enables organised and coordinated movement of groups of cells (morphogenesis) necessary for the formation and optimal dimensions of epithelial branches. In *Scrib*^*Crc/Crc*^ mutants, lung morphogenesis is disrupted, similar to the lungs of other PCP mutants, including *Celsr1*, *Vangl2* and *PTK7* (Paudyal et al., 2010; Yates et al., 2010). We have shown a genetic interaction between *Scrib* and *Vangl2* which leads to lung defects similar to those observed in homozygous mutants of either gene i.e., disordered epithelial organisation in airways and narrow or absent lumina. Consistent with published data in other tissues, we show that the *Scrib*^*Crc/Crc*^ mutation affects the localisation of the core PCP proteins Vangl2 and Celsr1 in the lung (Courbard et al., 2009; Montcouquiol and Kelley, 2003). Moreover, our data indicates Scrib operates via Rho signalling in the lung as a significant reduction in active RhoA was observed in *Scrib*^*Crc/Crc*^ lungs. Furthermore, *Scrib*^*Crc/Crc*^ mutant lungs show severe disruption to the actin–myosin cytoskeleton, which is the target of the PCP signalling pathway. Our data show that A/B polarity is largely unaffected in *Scrib*^*Crc/Crc*^ lungs, although we cannot completely rule out subtle alterations. Taken together, these data provide evidence that Scrib operates within the PCP pathway to regulate lung development.

### *Scrib* is required to maintain normal cell–cell contact and epithelial junctions

There are clear similarities between some of the phenotypes observed in *Scrib*^*Crc/Crc*^ lungs and those of Celsr1 and Vangl2 mutants, as would be expected from components of the same signalling pathway. However, *Scrib*^*Crc/Crc*^ lungs are more severely affected than *Vangl2*^*Lp/Lp*^ and *Celsr1*^*Crsh/Crsh*^ lungs (Yates et al., 2010). Macroscopically, *Scrib*^*Crc/Crc*^ lungs are smaller, with profound epithelial organisation defects and most ‘tubes’ lack a lumen. In *Celsr1* and *Vangl2* mutant lungs, most epithelial tubes have a visible lumen, although frequently, their diameter is considerably narrower than in wildtype lungs. All three of these mouse mutants display actin–myosin cytoskeletal defects that affect the overall structure and morphogenesis of lung epithelia.

Scrib also has distinct functions not shared with Celsr1 and Vangl2. *Scrib*^*Crc/Crc*^ lungs display defects in cell junctions and reduced cohesion of epithelial cells that are not observed in *Celsr1* or *Vangl2* mutants. Real-time imaging of *ex vivo Scrib* morpholino treated lung explant culture revealed reduced cell–cell contact and increased movement of epithelial cells, suggesting that cell adhesion might be affected. Organotypic cultures of *Scrib* morpholino treated lung cells also highlight the requirement for Scrib function in epithelial organization and lumen formation. Close epithelial cell–cell contacts, maintained by adherens and tight junctions, are critical for tubulogenesis and a pre-requisite for a correctly sized and positioned lumen at the centre of an airway (Affolter and Zeller, 2009; Andrew and Ewald, 2009; Morrisey and Hogan, 2010).

Previous studies have shown co-localisation between Scrib and the junctional proteins β-catenin and E-cadherin, and Scrib has been shown to influence recruitment and localisation of adherens junction proteins *in vitro* (Kamei et al., 2007; Navarro et al., 2005; Sun et al., 2009). Here we provide evidence that Scrib interacts with β-catenin in endogenous lung tissue. Consistent with such an interaction, the sub-cellular localisation of β-catenin is perturbed in *Scrib*^*Crc/Crc*^ lungs.

The interaction between β-catenin and Scrib appears to be specific because E-cadherin localisation was not notably changed in *Scrib*^*Crc/Crc*^ lungs. Structurally, β-catenin is an intracellular protein whereas E-cadherin contains both extra- and intra-cellular domains. Moreover, E-cadherin is involved in very early aspects of adherens junction formation, becoming stabilised at the cell surface where as β-catenin is subsequently recruited along with other proteins to form adherens junction complexes (Hartsock and Nelson, 2008). Importantly, β-catenin directly links the junctional complexes in the plasma membrane with the intracellular cytoskeleton, and this connection considerably strengthens cadherin-mediated adhesion (Yap et al., 1997). In addition to β-catenin mislocalisation, we also observed cytoskeletal disruption in *Scrib*^*Crc/Crc*^ lung epithelia, both of which are likely to contribute to destabilisation of the epithelial cells. Thus, differences in the functions of β-catenin and E-cadherin may explain why one and not the other is disrupted in *Circletail*.

Our immunochemistry studies show that Scrib protein is enriched in the tight junctions of lung epithelial cells, in addition to its localization to the plasma membrane. Tight junctions contribute significantly to epithelial cell stability and intact adherens junctions are believed to be required for tight junction formation (Yoshihara et al., 2011). Scrib protein is composed of multiple domains that mediate protein–protein interactions (Kallay et al., 2006; Metais et al., 2005; Petit et al., 2005; Sun et al., 2009). *In vitro*, Scrib has been shown to interact with ZO-1 and ZO-2 and the interaction with ZO-2 relies on PDZ domains 3 and 4 of Scrib (Ivanov et al., 2010a; Metais et al., 2005). Interestingly, our studies show that ZO-2 is spatially disrupted in *Circletail* mutants in which a premature stop codon results in a truncated protein lacking PDZ domains 3 and 4 (Murdoch et al., 2003). This domain-specific interaction between Scrib and ZO-2 may explain why ZO-2 localization was perturbed, whereas that of ZO-1 was largely unaffected in *Scrib*^*Crc/Crc*^ lungs. Since TEM analysis of tight junctions showed no obvious physical defect in tight junctions of *Scrib*^*Crc/Crc*^ lungs, and the overall levels of tight junction proteins were unaltered, this suggests that the destabilisation of epithelial cells is likely due to mislocalisation of specific proteins within the junctional complexes, rather than an absence or mechanical separation of tight junctions. Taken together with the multi-layered disorganized epithelium, severe reduction of visible lumina and increased cell density in *Scrib*^*Crc/Crc*^ airways, we propose that disruption of Scrib leads to reduced cohesion between epithelial cells, causing destabilization of the epithelium and filling of the luminal space. Since a large proportion of the normal lung volume is air space, this loss of luminal space leads to a considerable reduction in overall lung size in *Scrib*^*Crc/Crc*^ mice. Further evidence of a role for Scrib in cell–cell cohesion comes from our analysis of de novo epithelial cyst formation in lung organotypic cultures. In these assays, Scrib knockdown results in visible cellular disorganisation and absence of a centrally located, expanded lumen as well as mislocalisation of β-catenin and ZO-2. These data are consistent with previous studies showing that Scrib can act as a tumour suppressor by helping to maintain epithelial organisation and that its expression is downregulated in a number of human cancers (Gardiol et al., 2006; Ivanov et al., 2010b; Navarro et al., 2005; Pearson et al., 2011; Thomas et al., 2005; Zhan et al., 2008).

Whilst the observation that there are intact tight junctions in the *Scrib*^*Crc/Crc*^ lungs seen by TEM may at first appear inconsistent with the reduced cohesion of epithelial cells in time-lapse imaging of ex vivo lung culture and mislocalisation of adherens and tight junction proteins, we propose that these data are consistent and in fact the mislocalisation of junctional proteins does not lead to a loss of adherens or tight junctions but instead results in weaker cell–cell interactions (reduced cohesion), allowing dynamic dissociation and subsequent re-formation of cellular junctions. This understanding of the data would not have been reached without the use of real-time imaging in 3D culture along with the more traditional 2D imaging techniques at single timepoints.

### The relationships between polarity pathways and lung branching morphogenesis

The importance of the PCP signalling pathway for the generation of glands and organs that undergo branching morphogenesis is beginning to be realised. A number of studies have shown that PCP genes are required for lung and kidney organogenesis (Carroll and Das, 2011; Yates and Dean, 2011). However, in the kidney, mutations in genes that disrupt the PCP pathway frequently lead to increased lumen diameter and cyst formation (Fischer et al., 2006; Karner et al., 2009). PCP function is also necessary in other branched structures like the lacrimal and salivary glands as morphogenetic defects in these tissues are observed in PCP mutant mice (C.D. unpublished observations).

**Fig. 9:**
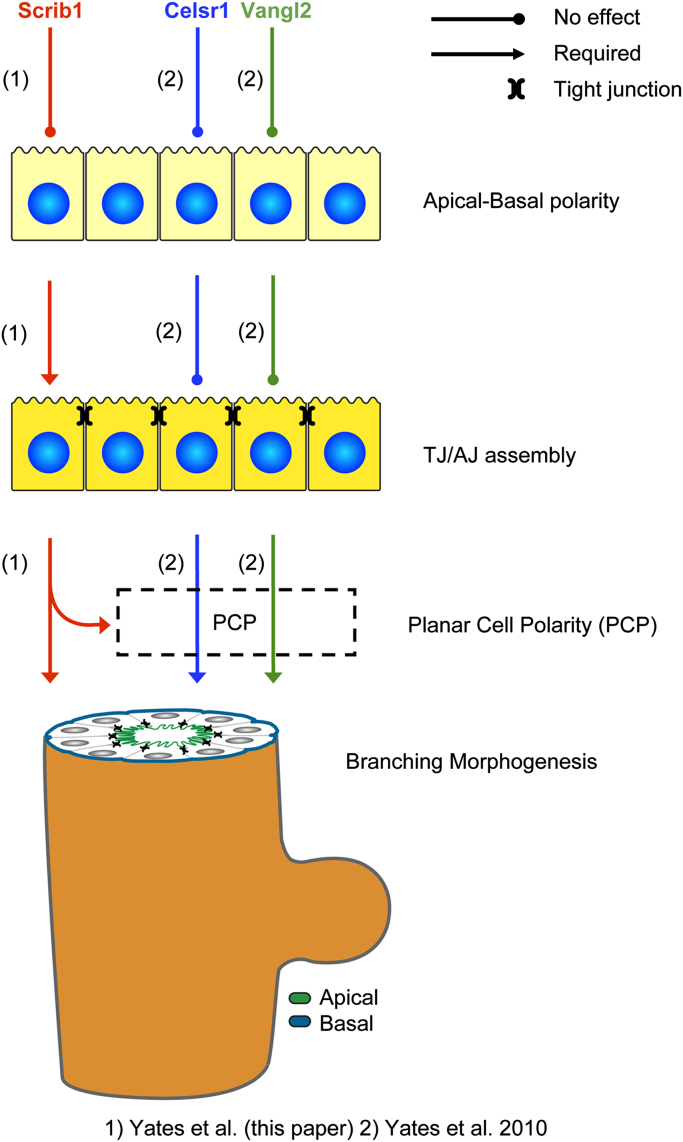
Model depicting the potential hierarchy between A/B polarity, planar polarity and branching morphogenesis in the lung. The relationships of *Scrib1*, *Celsr1* and *Vangl2* to these processes are indicated (based on the results described here and in Yates et al., 2010).

Our investigations into the roles of Celsr1, Vangl2 and Scrib in lung development have allowed us to build an understanding of the relationships between planar polarity, A/B polarity and tight junction formation in lung development (summarised in Fig. 9). Biophysically, these three cellular mechanisms could occur simultaneously and one or more aspects be mediated by the same protein. Our studies indicate that *Celsr1*, *Vangl2* and *Scrib* are all required for planar polarity and lung branching morphogenesis, However, none of the studied alleles show a disruption in A/B polarity, suggesting that A/B polarity and planar polarity are separate steps required for branching morphogenesis. Tight junction formation may also be a separable step because Scrib loss affects the sub-cellular organisation of tight junction proteins, presumably leading to their disruption, yet these alterations do not appear to affect other A/B polarity markers. This, and other studies, lead us to conclude that A/B polarity is a prerequisite for tight junction assembly (Umeda et al., 2006) and further that tight junction assembly is necessary for branching morphogenesis to occur (Chung and Andrew, 2008; Feigin and Muthuswamy, 2009; Hogan and Kolodziej, 2002; Martin-Belmonte and Mostov, 2008). We have shown that *Scrib*, like *Celsr1* and *Vangl2*, is required for PCP pathway function, and PCP signalling is necessary for lung branching morphogenesis. Notably though, this and our previous studies have shown that in addition to the similarities in function between Celsr1, Vangl2 and Scrib, there are also clear differences. In particular, we have previously shown that Celsr1 is important for bud bifurcation in lung branching (Yates et al., 2010) whereas Scrib does not affect bifurcation and instead is required for proper epithelial cell cohesion (Fig. 4 and Movies 1 and 2).

Our results reveal an important role for *Scrib* in aspects of lung development, specifically, in formation and maintenance of junctional complexes and in lumen morphogenesis. These data broaden our understanding of the mechanisms of lung development and will likely provide important insight into the pathobiology of diseases featuring abnormal epithelial organisation and lumen morphology.

## Footnotes

Appendix A Supplementary data associated with this article can be found in the online version at 10.1016/j.ydbio.2012.11.012.
